# Epidemiological Study of Road Traffic Accident Cases from Western Nepal

**DOI:** 10.4103/0970-0218.62568

**Published:** 2010-01

**Authors:** Badrinarayan Mishra, Nidhi D Sinha (Mishra), SK Sukhla, AK Sinha

**Affiliations:** Rural Medical College, Loni, India; 1 Rural Dental College, RMC, Loni, India; 2 Department of Medical Informatics, RMC, Loni, India; 3 Department of Orthopedics, Manipal College of Medical Sciences, Pokhara, Nepal

**Keywords:** Epidemiological factors, out comes, road and traffic accidents, western Nepal

## Abstract

**Background:**

Road Traffic Accident (RTA) is one among the top five causes of morbidity and mortality in South-East Asian countries.(1) Its socioeconomic repercussions are a matter of great concern. Efficient addressing of the issue requires quality information on different causative factors.

**Research Question:**

What are different epidemiological determinants of RTA in western Nepal?

**Objective:**

To examine the factors associated with RTA.

**Study Design:**

Prospective observational.

**Setting:**

Study was performed in a tertiary healthcare delivery institute in western Nepal.

**Participants:**

360 victims of RTA who reported to Manipal Teaching hospital in one year.

**Study Variables:**

Demographic, human, vehicular, environmental and time factors. Statistical analysis: Percentages, linear and logarithmic trend and Chi-square.

**Results:**

Most of the victims i.e. 147 (40.83%) were young (15 to 30 years); from low i.e. 114 (31.66%) and mid i.e. 198 (55%) income families and were passengers i.e. 153 (42.50%) and pedestrians i.e. 105 (29.16%). Sever accidents leading to fatal outcome were associated with personal problems (*P*<0.01, χ^2^ - 8.03), recent or on-day conflicts (*P*<0.001, χ^2^ - 18.88) and some evidence of alcohol consumptions (*P*<0.001, χ^2^ - 30.25). Increased prevalence of RTA was also noticed at beginning i.e. 198 (55%) and end i.e. 69 (19.16%) of journey; in rainy and cloudy conditions (269 i.e. 74.72%) and in evening hours (3 to 7 p.m. 159 i.e. 44.16%). Out of 246 vehicles involved; 162 (65.85%) were old and ill maintained. The contributions of old vehicle to fatal injuries were 33 (50%). Head injury was found in 156 (43.33 %) cases and its associated case fatality rate was 90.90%. In spite of a good percentage receiving first aid i.e. 213 (59.16%) after RTA; there was a notable delay (174 i.e. 48.33% admitted after 6 h) in shifting the cases to the hospitals. The estimated total days lost due to hospital stay was 4620 with an average of 12.83 days per each case.

**Conclusion:**

Most of the factors responsible for RTA and its fatal consequences are preventable. A comprehensive multipronged approach can mitigate most of them.

## Introduction

The statistical profile reflects a global estimate of 5.1 million deaths in 2000, which was due to injuries that accounted for 10% of deaths due to all causes. Out of this a quarter of injury-related deaths occurred in the South-East Asian region.(2) In fact, road traffic injuries alone ranked as the number one cause of disease burden among children between 5 and 14 years, and as the number three cause among those in the age group 15 to 29 years with a male female ratio of 3: 1.(3,4) Most commonly affected road users are pedestrians, passengers and cyclists as opposed to drivers who are involved in most of the deaths and disabilities. This ever expanding epidemic targeting the young and productive generations is likely to take a heavy burden on the quality of life and socioeconomic growth of the region.(5)

Pokhara city, the focus of present study, has some geopolitical distinctions. It is situated in central Nepal; located at 28.24°N, 83.99°E and 198 km west of Kathmandu. It spans 8 km from north to south and 6 km from east to west. Unlike Kathmandu, it is quite loosely built up. This is the Headquarter of Kaski district, the Gandaki zone and Western Development Region of Nepal. It is also one of the most popular tourist destinations in Nepal. The total area of Kaski district including Pokhara city is 2017 km^2^ and has a population of 3,80,527. In no other place the mountains rise so quickly, within 30 km, from 1000 to over 8000 m. The Dhaulagiri, Annapurna and Manaslu ranges, each with peaks over 8000 m are within striking distance from Pokhara. Due to this sharp rise in altitude, the area of Pokhara has one of the highest precipitation rates of the country (over 4000 mm/year). The climate is sub-tropical but due to the elevation the temperatures are moderate; the summer temperatures average between 25 and 35°C, in winter around 5 and 15°C.(6–8)

From Nepal's prospective, the deep rooting of RTA has few additional factors such as the difficult terrain, political instability, night time unofficial curfew imposed by Maoist guerillas and universal availability of alcohol. In this land-locked Himalayan kingdom, injuries accounted for 2% of hospital admissions, occupying the ninth leading position. They also accounted for nearly 60–70% of emergency room registrations at major tertiary care hospitals. Road Traffic Accident (RTA) is the number one cause (80 to 90%) for all injuries.(2–5)

The World Health Organization (WHO) in its international conference on RTA noted the importance of adequate data on traffic injuries. Indeed, accurate estimates of the public health burden of RTA can establish the priority of this public health problem, and provide a rational basis for policy decisions.(7–9)

Despite this, there is a little recognition of the health and economic burden of this problem. Studies on RTA are far and few in Nepal. Surprisingly, Pokhara being the second largest city and a tourist heartthrob has no epidemiological study on RTA. The geographical complexity of the region appears to make it a place of special interest. The backdrop this study, the first of its kind in Pokhara, was planned to determine the epidemiological determinants of RTA.

## Materials and Methods

### Place of study

This descriptive study was conducted at Manipal Teaching Hospital (MTH); the only referral tertiary care teaching hospital of Manipal College of Medical Sciences (MCOMS) situated in the south central part of the Pokhara city.(4,7)

### Duration and type of study

This descriptive study was conducted from 1st June 2004 to 31^st^ May 2005.

### Study group

It consisted of all the RTA victims reporting to MTH in the above one year period.

### Case definition

For the purpose of the study, an RTA was defined as an accident, which took place on the road between two or more objects, in which one is any kind of moving vehicle and the other a human being.

### Case selection and exclusion

The victims/relatives of the RTA cases, who attained either the casualty, orthopedic outpatient clinic, surgical outpatient clinic or admitted in the wards of MTH, were interviewed to obtain the information about the circumstances leading to the accident. The victims and relatives (in case of unconscious patients) who did not consent to be a part of the study were excluded.

### Data collection

A pre-tested proforma specially designed for this purpose was used. The information collected consisted of general epidemiological data, category of road users, day and time of accident, severity of injuries and treatment outcome. The medicolegal records and case-sheets of the victims were referred for collecting additional information and where necessary for cross-checking.

## Results

### Sociodemographic factors and RTA

Age, sex and religion break ups: Among 360 RTA victims, most cases 138 (38.33%) were in the age group of 15–30 years. A high percentage of both fatal 30 cases out of total 66 (45.45%) and non-fatal 108 out of total 294 (36.73%) cases were observed from the same age group. Mobile males (85%) outscored the domicile females (15%) with a ratio of 5.66:1. Hindus and Buddhists dominated the study population i.e. 216 (60%) and 138 (38.33%), respectively as per the expected religion breakup.(10,11)

Family type, residence, education and socioeconomic breakups: Most of the cases were from joint family i.e. 247 (68.61%). Victims from rural areas 237 (65.83%) were more as compared to urban areas 123 (34.17%). High prevalence of RTA was reported in school educated i.e. 177 (49.16%) and graduates i.e. 141 (39.15%). People from middle and low socioeconomic class were also affected more i.e. 198 (55%) and 114 (31.66%), respectively.

The detailed demographic profile is presented in Table 1.

**Table 1:** Demographic profile of RTA victims

Demographic criteria	No.of cases	Percentage
Age		
0-15	42	11.67
16-30	147	40.83
31-45	87	24.17
46-60	51	14.17
>60	33	9.17
Total	360	100.00
Sex		
Female	54	15.00
Male	306	85.00
Total	360	100.00
Marital status		
Married	174	48.33
Single	186	51.67
Total	360	100.00
Religion		
Hindu	216	60.00
Buddhist	138	38.33
Christia/Muslims	06	01.66
Total	360	100.00
Family		
Nuclear	123	34.17
Joint	237	65.83
Total	360	100.00
Residence		
Rural	237	65.83
Urban	123	34.17
Total	360	100.00
Education		
School	171	47.50
Graduate	141	39.17
Illiterate	48	13.33
Total	360	100.00
Socioeconomic status		
Middle	198	55.00
Lower	114	31.67
Upper	48	13.33
Total	360	100.00

### Human factor in RTA

Personal history and RTA: Personal problems like deviance, hyperactivity, low tolerance, inattentiveness were associated with 120 (33.33%) RTA cases. The presence of personal problems was found to be significantly associated with fatality (*P*<0.01, χ^2^ − 8.03).

Alcohol consumption and RTA: ‘Alcohol - the killer on road’ is a welldocumented fact.(12–17) A victim with smell of alcohol from breath or mouth±clinical evidence in form of motor incoordination and verbal aberration was considered for some evidence of alcohol consumption. Out of 69 drivers sustaining RTA in the present study, 32 (46.37%) were found to have some evidence of alcohol consumption, and out of the 32 with these evidence 27 (84.37%) succumbed to their injury. The χ^2^ value for this was found to be 30.25 (*P*<0.001).

Disease conditions and RTA: Out of the total study population, 30 (8.33%) were found to have disease conditions like diabetes, hypertension, tuberculosis. Those who had chronic diseases were significantly associated with high fatality. χ^2^=7.31; *P*<0.01. Table 2 gives the details of these observations.

**Table 2:** Disease condition and severity of accident in RTA cases

Disease condition	Non-fatal	Fatal	Total
Present	15	15	30
Absent	279	51	330
Total	294	66	360

X^2^ = 7.31;

*P* < 0.01

Psychosocial conflicts: History of recent or on-day conflict was found in 39 (10.83%) of cases. But in the fatally injured category, a staggering 24 (61.53%) had some form of conflicts prior to accident. Presence of conflict was found to be significantly associated with fatality (*P*<0.001, χ^2^-18.88).

### Types of victims and RTA

Road users in RTA: Out of the total 360 cases, 153 (42.50%) were passengers, 105 (29.16%) pedestrians, 69 (19.17%) drivers and 33 (9.61%) cyclists. The passenger and pedestrian category was found to be involved mainly in between 3 and 7 pm i.e. 123 (47.67%) and 7–to 11 am i.e. 51 (19.76%). The time period in which minimum number of cases (i.e. 0 cases) was reported is from 11 pm to 3 am.

Drivers and RTA: Out of the total 360 RTA cases, 69 (19.16 %) were drivers. The hour of the day when this group was involved most was from 3 to 7 pm i.e. 24 (34.78%). The time of involvement again differs from other studies.(16–22)

Cyclists and RTA: Total number of cyclists involved in RTA in the present study was 33 (9.61%). The pick hour for the cyclist to get involved in RTA was from 7 to 11 a.m. i.e.15 (45.45 %) and 3 to 7 p.m. i.e. 12 (36.36%).

### Time factors in RTA

Time of accident: Maximum number of accidents occurred in between 3 and 7 p.m. i.e. 159 (44.16%) followed by 87 (24.16%) between 7 and 11 a.m. Mortality wise highest number of cases were found between 3 and 11 p.m. at a percentage of 48 (72.72%). The distributions of non-fatal cases were maximum from 3 to 7 p.m. i.e. 132 (44.89%).

Weekdays and RTA: A total of 201 accidents happened during week days. Here two distinct picks were found, i.e. between 3 to 7 p.m. and 7 to 11 a.m. at 81 (40.29%) and 66 (32.83%), respectively.

Weekends and RTA: For weekends 3 to 7 p.m. recorded the maximum cases i.e.72 (45.28%) out of the total 159.

**Figure 1:**
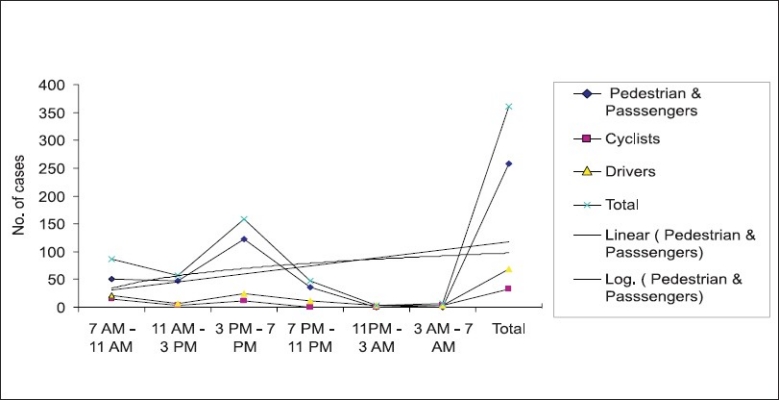
Highlights the positive trend both linear and logarithmic for pedestrians and passenger category

Figure 1 highlights the positive trend both linear and logarithmic for pedestrians and passenger category.

### Environmental factors and RTA

Weather conditions and RTA: From the present study it was observed that 269 (81.66%) RTA occurred in rainy and cloudy conditions.

**Figure 2:**
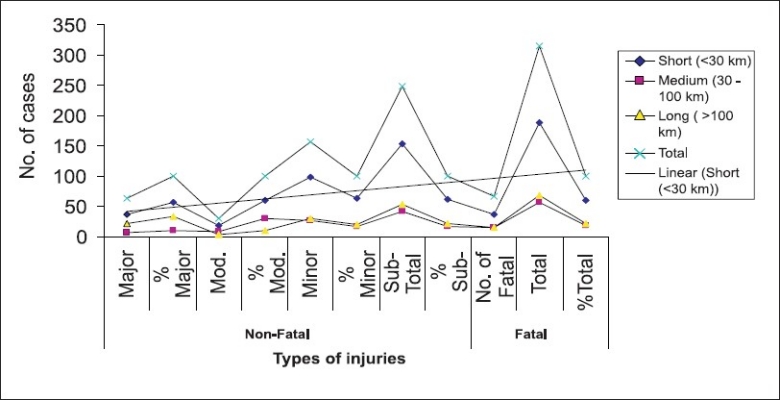
Distance traveled and frequency of different types of injuries

Distance covered and RTA: Most of the accidents occurred at the starting of the journey i.e. 198 (55%) and the trend again picked at the fag end of the journey i.e. 69 (19.16%). Death due to accident also showed a similar trend with high prevalence at starting i.e. 36 (54.54%) and 24 (36.36%) at fag end. Figure 2 shows the frequency of different types of injuries to that of the distance traveled. A linear trend is noticed between different types of injuries during the initial part of the journey (<30 km.).

Speed of the vehicle and RTA: It was observed that 180 (50%) cases were due to high speeding vehicles (40–60 km/h.). speeding vehicles were responsible for an alaramingly high percentage of fatal accidents i.e. 42 (63.63%). The speed limit of 40–60 km/h is considered high, keeping in mind the difficult terrain and geographical complexities.

### Road condition and RTA

Narrow and wide: According to the study, 213 (59.16%) of accidents were collision type and 147 (40.83%) were non-collision type. Narrow and defective roads were responsible for 39 (26.53%) of non-collision accidents where as collision types occurred mostly in wide roads i.e. 132 (61.97%). Non-collision accidents like running of the road, overturning, knocking down of pedestrians were found to be significantly associated with narrow and defective road conditions (*P* < 0.001, χ^2^-20.21).

Familiar and non-familiar: About 306 (85%) of RTA occurred in familiar roads to that of 51 (14.17%) in non-familiar ones. The collision and non-collision types in familiar road conditions were 174 (56.86%) and 135 (44.11%), respectively. But in non-familiar road conditions, collision type was at a staggering 76.47% i.e. 13 out of 17 RTA.

**Figure 3:**
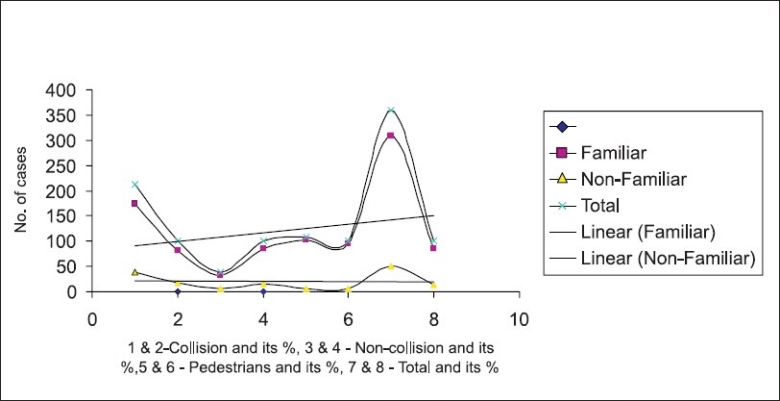
Types of RTA and road familiarity

Figure 3 shows types of RTA in relation to familiarity to road conditions. A linear trend was noticed for different types of accidents in familiar roads; so also a positive trend was noticed for accidents among pedestrians. Surprisingly no (0) trend was noticed for any types of RTA in non-familiar roads.

### Traffic conditions and RTA

Traffic police: Out of 360 cases requiring admission to the hospital, only 21 (5.83%) occurred in presence of the traffic police. The mortality and morbidity percentage were similarly low at 3 cases (4.55%) and 18 cases at (6.12%) with a significant *P*<0.001.

Traffic light: Only 18 (5%) cases of RTA requiring hospital visit occurred in the presence of effective traffic light. The mortality and morbidity percentage were similarly low at 3 cases (4.55%) and 15 cases at (5.10%) with a significant *P* < 0.001. In fact, there was a slight advantage for the presence of traffic light over the presence of only traffic police in averting RTA.

### Vehicle condition

Age of the vehicle: Out of the total 246 vehicles involved, 162 (65.85%) were old and 84 (34.14%) were new. Out of 42 fatal accidents caused by motor vehicles, 33 (78.57%) were due to old ones. Old vehicles were also responsible for majority of non-fatal accidents i.e. 129 (43.67%). A positive trend (linear) was observed with old vehicle in respect to both fatal and non-fatal RTAs.

Vehicular accessories: Out of the total 246 vehicles involved, 54 (21.95%) were without any accessories.

### Victim condition and RTA

Site of injury: From 360 RTA cases 156 (43.32%) had head injury. Out of 66 fatal cases, 60 (90.90%) sustained head injury. In the group of 204 RTA cases without head injuries, 198 (97.05%) were non-fatally injured. The association of fatality was very significantly associated with the presence of head injury at the time of accident (*P* < 0.001).

First-aid and RTA: The first-aid coverage in the present study was 213 (59.16%); however, the coverage for fatally injured was at a low at 45.45% (30 victims) in comparison to non-fatally injured at 62.24% (183 cases). The detailed break up is presented in Table 3.

**Table 3:** First-aid and severity of accident in RTA cases

First-aid	Non-Fatal								Fatal			
		
	Major	% Major	Moderate	% Moderate	Minor	% Minor	Sub-total	% Sub-total	No. fatal	% Fatal	Total	% Grand total
Given	39	61.90	18	50.00	126	64.62	183	62.24	30	45.45	213	59.17
Not-given	24	38.10	18	50.00	69	35.38	111	37.76	36	54.55	147	40.83
Total	63	100.00	36	100.00	195	100.00	294	100.00	66	100.00	360	100.00

**Figure 4:**
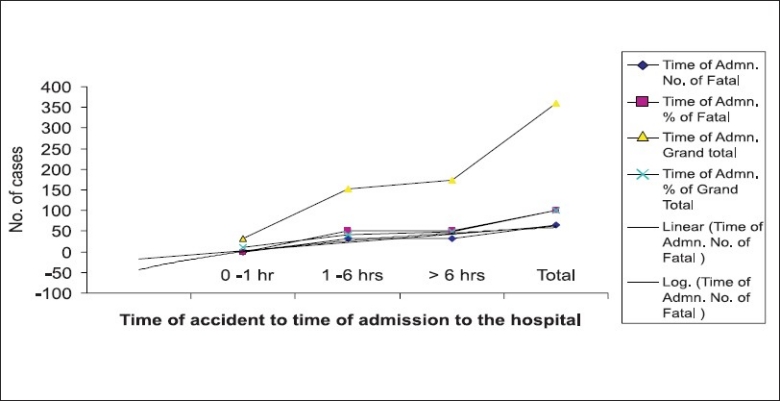
Trend of RTA outcome in relation to admission to the hospital

Time of accident and RTA admission: Only 10% (36 cases) were admitted to the hospital within one hour, 41.67% (150 cases) between 1 and 6 hours and the majority of cases beyond 6 hours i.e. 174 (48.33 %). of accident. Figure 4 shows the trend of RTA outcome in relation to admission to the hospital. A positive linear trend as well as logarithmic trend was observed with delay in admission to the hospital and severity of outcome.

Days lost due to RTA: The estimated total days lost in the present study was 4620 days in the form of hospital stay with an average of 12.83 days per each case. The longest period was recorded to be 90 days and the shortest 1 day.

## Discussions

We would like to conclude the findings of the present study under the following broad categories.

### Sociodemographic factors

The present study revealed most victims were young, unmarried, pedestrians and drunk drivers. On socio-educational front, majority of them were educated up to school and belonged to middle and lower socioeconomic classes. Similar observations were made by researchers from neighboring countries including India.(4,10–12,15,17) This situation can be improved by educating public through the mass media and initiating road safety training campaign in schools. This should highlight the socioeconomic and other problems of RTA and the role of individuals in its prevention. The association of alcohol consumption is related to free availability and non-restriction on sale. It is time for the Government to learn from other country and have some strong legal enforcement.

Medical conditions, personal problems and psychosocial conflicts were found to be significantly association with RTA. The strong association of these factors is well-documented world over.(17–21) The existing sociopolitical turmoil and the consequent uncertainty and instability of the country has likely contributed to increased psychosocial and interpersonal conflicts. An early address of this problem is urgently required to bring down the general stress level and to effectively contribute to social and economic stability.

### Vehicular factors

More of fatal and nonfatal accidents were reported with over-aged ill-maintained vehicles. Roadworthiness of plying vehicles is an important factor in RTA prevention.(22–25) Certifying the roadworthiness of the vehicle should be legitimized. Any vehicle found lacking in roadworthiness should not be permitted for plying.

Speeding vehicle and long distance travel were found to be associated with high percentage of RTA. Accidents were also high in the present series at the start of journey as well as at the fag end of the journey. An effective speed policies for different zones along with scanning for speedy and rash driving through speed detecting camera at strategic and accident black spots together with timely refresher courses for the drivers are some of the activities where the government and local bodies should focus upon.(11–13,26–29)

### Organizational factors

Narrow and defective roads had the majorities of RTA in our study. Ply-worthy roads are a prerequisite for effective RTA prevention.(10–12,25–28) So there is a need of timely road maintenance, proper road engineering with sufficient traffic signs which can go a long way in improving the road worthiness of the roads.

The study demonstrated that the presence of traffic police and traffic light had a significant impact on the number and severity of accident. In fact, presence of functioning traffic light showed better accident control than the presence of traffic police alone. An educated traffic responds better to traffic police than lights and signals are being documented widely.(12,16,28) The reverse observation may be linked to the indulgence of traffic police in petty corruption at traffic crossings. We suggest the employment of more and more traffic police and traffic signals at road crossings and accident black spots. The tract record of the manning police should also improve.

Accident investigation is one of the gray areas in RTA studies. In our series we did not find any single instance where the accident dynamic was studied to evaluate the impact and extent of the damage.

Encouragingly, availability of first-aid services was found in good number of cases. The reported services were more out of compassion and fellow felling. The quality of service was found lacking as most of the providers were untrained. Similar situations prevail in most South-East Asian countries.(16–19,21,30) The government should try to encash on this positive public perception to improve both quantitative and qualitative first-aid services by training local volunteers.

Last but not the least, the timing of admission of cases to hospital is crucial in saving the life of the victim.(28–30) In the present study it was observed that there was a dramatic improvement in outcome of victims admitted to the hospital within one hour of accident. So an effort should be made to provide timely and proper medical services to RTA victims via mobile emergency services, quality trauma centers and proper rehabilitation services.

### Recommendation

Interventions in TRA are broad-based and include regulation, legislation and community projects. The government of Nepal should find ways to support policy at local level. This should be client-oriented with good community support so as to overcome limitations. The focus should be on issues like establishment of Provincial Safety Committee, Motorcycle Helmet Campaign, Anti-Drunk-Driving Campaign, Traffic Injury Prevention in School, Safe Communities and establishment of trauma registry and The Pre-hospital Care System, which are proven ways of tackling RTA. A huge potential exists for such an approach. Public health alone cannot win the war against RTA. The public health sector can play an important role in taking the lead in advocacy and support; it can add value to epidemiology and information systems, among others. However, partnerships need to be formed with public, private and non-governmental organizations to address more visibly the problems and press harder for improvements. Political leadership, good governance, policy support and a reliable technical team are the key components on which Nepal should be zeroing in.
